# Do patients with oxyphilic cell papillary thyroid carcinoma have a poor prognosis? Analysis of the surveillance, epidemiology, and end results database 2004-2013 with propensity score matching

**DOI:** 10.18632/oncotarget.20355

**Published:** 2017-08-18

**Authors:** Chunping Liu, Qiuyang Zhao, Wen Zeng, Chen Chen, Jie Ming, Shuntao Wang, Yiquan Xiong, Chao Zhang, Tianwen Chen, Zeming Liu, Tao Huang

**Affiliations:** ^1^ Department of Breast and Thyroid Surgery, Union Hospital, Tongji Medical College, Huazhong University of Science and Technology, Wuhan 430022, China; ^2^ Department of Ophthalmology, Zhongnan Hospital, Wuhan University, Wuhan, China; ^3^ Department of Cardiovascular Surgery, Union Hospital, Tongji Medical College, Huazhong University of Science and Technology, Wuhan, China; ^4^ Department of Breast and Thyroid Surgery, Affiliated Nanshan Hospital, Guangdong Medical University, Shenzhen, China

**Keywords:** oxyphilic cell papillary thyroid carcinoma, prognosis, SEER, PSM

## Abstract

The prognosis of oxyphilic cell papillary thyroid carcinoma (OCPTC) remains unclear. The aim of this study was to investigate the prognosis of OCPTC and provide a new perspective on treatment guidelines for these patients. We investigated a large cohort of DTC patients from the Surveillance, Epidemiology, and End Results (SEER) database between 2004 and 2013. Patient mortality was examined by Kaplan-Meier analyses with log-rank tests and Cox proportional hazards regression analyses. In the study cohort, the rate of cancer-specific mortality per 1000 person-years for OCPTC was lower than that for classic papillary thyroid cancer (CPTC) and follicular thyroid cancer (FTC). According to the multivariate Cox regression model, the cancer-specific and all-cause mortality rates of OCPTC were similar to that of CPTC and FTC. The cancer-specific survival rate in patients with OCPTC was higher than that in patients with FTC, but similar to patients with CPTC, after matching for influential factors using propensity score matching analysis. The unanticipated prognosis provided new implications for the treatment of patients with OCPTC.

## INTRODUCTION

Thyroid cancer has been rising rapidly in recent decades [1–5]. Papillary thyroid cancer (PTC) accounts for 80–90% of all thyroid malignancies, making it the most common type of thyroid malignancy [6]. Rare histological variants of PTC include follicular, tall cell, columnar cell, diffuse sclerosing, solid, hobnail, and insular variants [7–11].

The Surveillance, Epidemiology, and End Results (SEER) program of the National Cancer Institute (NCI) is the largest publicly available data source for cancer incidence and survival in the United States [12, 13]. Propensity score matching (PSM) method is a statistical matching technique for analyzing observational data by estimating the effects of a treatment, policy, or other intervention and accounting for covariates that predict receiving the treatment. PSM attempts to reduce the bias due to confounding variables.

Hürthle cell tumors in thyroid neoplasms include adenomas, carcinomas, and papillary thyroid carcinomas [i.e., oxyphilic cell papillary thyroid carcinoma (OCPTC)]. Hürthle cell carcinoma a rare variant of PTC and considered an uncommon and more aggressive thyroid cancer by many researchers [14, 15]. However, there is still a lack of research about OCPTC, especially in large populations. In this study, we investigated the prognosis of OCPTC on the basis of reliable and large-scale research dataset from the SEER database 2004–2013 using PSM methods.

## RESULTS

### Demographic and clinical features

A total of 66305 patients with different histological subtypes [n = 147, OCPTC; n = 60739, classic papillary thyroid cancer (CPTC); and n = 5419, follicular thyroid cancer (FTC)] were included in this study. The study patients’ mean age and survival in months for the different histological subtype stages are shown in Table 1. Patients with OCPTC had significantly shorter months of survival than patients with other stages.

**Table 1:** Characteristics for Patients with different histological types

Covariate	level	Histological types				
		OCPTC (n=147)	CPTC (n=60739)	p-value	FTC (n=5419)	p-value
Age (year)		52.22±16.46	48.36±15.35	0.001	50.79±17.29	0.253
Sex	Female	109(74.1%)	46786(77.0%)	0.407	3843(70.9%)	0.394
	Male	38(25.9%)	13953(23.0%)	1576(29.1%)		
Race	White	131(90.3%)	49651(82.8%)	0.056	4186(78.3%)	0.001
	Black	4(2.8%)	3159(5.3%)	640(12.0%)		
	Other	10(6.9%)	7133(11.9%)	517(9.7%)		
T stage	T1	77(52.7%)	37974(63.8%)	0.001	1240(23.7%)	<0.001
	T2	35(24.0%)	8062(13.6%)	2110(40.4%)		
	T3	31(21.2%)	10845(18.2%)	1682(32.2%)		
	T4	3(2.1%)	2599(4.4%)	191(3.7%)		
N-stage	N0	118(82.5%)	44102(74.9%)	0.001	5114(96.9%)	<0.001
	N1	25(17.5%)	14744(25.1%)	161(3.1%)		
M-stage	M0	144(%)	59951(%)	0.427	5093(%)	0.044
	M1	3(%)	788(%)	326(%)		
Multifocality	No	87(62.1%)	35549(60.1%)	0.624	4464(85.7%)	<0.001
	Yes	53(37.9%)	23591(39.9%)	742(14.3%)		
Extension	No	121(82.3%)	49129(82.1%)	0.935	4795(90.4%)	0.001
	Yes	26(17.7%)	10744(17.9%)	512(9.6%)		
Radiation	None or refused	70(48.6%)	30701(51.7%)	0.112	2303(43.6%)	0.309
	External beamradiation therapy	6(4.2%)	1105(1.9%)	163(43.6%)		
	Radioactive I-131 ablation	68(47.2%)	27548(46.4%)	2822(53.4%)		
Surgery	Biopsy	1(0.7%)	1513(2.5%)	0.432	183(3.4%)	0.317
	Lobectomy	20(13.9%)	7750(12.9%)	1207(22.5%)		
	Subtotal or near-total thyroidectomy	7(4.9%)	2116(3.5%)	277(5.2%)		
	Total thyroidectomy	116(80.6%)	48771(81.1%)	5363(68.9%)		
Survival months(month)		43.59±33.60	48.98±33.40	0.038	52.67±33.48	0.001

OCPTC: oxyphilic cell papillary thyroid carcinoma; CPTC: classic papillary thyroid cancer; FTC: follicular thyroid carcinoma;

### Cancer-specific and all-cause mortality rates for different histological subtypes

In the study cohort, the cancer-specific mortality rate, per 1000 person-years, for OCPTC, CPTC, and FTC were 1.872 [95% confidence interval (CI), 0.264–13.293], 2.512 (95% CI, 2.323–2.718), and 6.68 (95% CI, 5.722–7.809), respectively (Table 2). The all-cause mortality, per 1000 person-years, in patients with OCPTC, CPTC, and FTC were 16.852 (95% CI, 8.769–32.389), 10.538 (95% CI, 10.141–10.950) and 18.583 (95% CI, 16.929–20.399), respectively (Table 2).

**Table 2:** Hazard Ratios of different histological types for the cancer specific deaths and all cause deaths of thyroid cancer

Histological types	Cancer-Specific Deaths,	%	Cancer-Specific Deaths per	95% CI	All Cause Deaths,	%	All Cause Deaths per	95% CI
	No.		1,000 Person-Years		No.		1,000 Person-Years	
OXPTC	1	0.68	1.872	0.264-13.293	9	6.12	16.852	8.769-32.389
CPTC	659	1.08	2.512	2.323-2.718	2722	4.48	10.538	10.141-10.950
FTC	178	3.28	6.685	5.722-7.809	474	8.75	18.583	16.929-20.399

OXPTC: oxyphilic cell papillary thyroid carcinoma; CPTC: classic papillary thyroid cancer; FTC: follicular thyroid carcinoma;

### Risk factors for thyroid cancer-specific and all-cause mortality rates

According to the univariate Cox regression analyses, age, sex, race, T/N/M stage, extension, radiation treatment, and surgical approach were significant risk factors of cancer-specific mortality. In the multivariate Cox regression model, CPTC and FTC showed no significant risk for cancer-specific mortality compared to OCPTC after adjusting for influential risk factors (Table 3). In the univariate Cox regression analyses, age, sex, race, TNM stage, multifocality, radiation, and surgical approach were found to be significant risk factors for all-cause mortality. In the multivariate Cox regression analysis, OCPTC showed no significant risk for all-cause mortality compared to CPTC and FTC (Table 3).

**Table 3:** Risk factors for survival: outcome of thyroid cancer specific Mortality and all-cause mortality

Covariate	level	Thyroid Cancer specific mortality				All cause mortality			
		Univariate Cox regression		Multivariate Cox regression		Univariate Cox regression		Multivariate Cox regression	
		Hazard Ratio (95% CI)	p-value	Hazard Ratio (95% CI)	p-value	Hazard Ratio (95% CI)	p-value	Hazard Ratio (95% CI)	p-value
Age		1.098(1.092-1.103)	<0.001	1.063(1.057-1.070)	<0.001	1.087(1.084-1.089)	<0.001	1.072(1.069-1.075)	<0.001
Sex	Female	ref		ref		ref		ref	
	Male	2.630(2.295-3.014)	<0.001	1.117(0.939-1.328)	0.212	2.422(2.258-2.597)	<0.001	1.580(1.485-1.713)	<0.001
Race	White	ref		ref		ref		ref	
	Black	1.157(0.874-1.530)	0.308	1.018(0.699-1.483)	0.925	1.334(1.170-1.521)	<0.001	1.255(1.078-1.462)	0.003
	Other	1.412(1.168-1.708)	<0.001	0.951(0.749-1.207)	0.678	0.929(0.828-1.042)	0.206	0.791(0.692-0.905)	0.001
histological types	OXPTC	ref		ref		ref		ref	
	CPTC	1.480(0.208-10.520)	0.695	0.747(0.105-5.332)	0.771	0.659(0.343-1.269)	0.212	0.744(0.334-1.659)	0.470
	FTC	4.289(0.601-30.615)	0.147	1.184(0.164-8.529)	0.867	1.206(0.623-2.331)	0.578	0.893(0.397-2.005)	0.783
T-stage	T1	ref		ref		ref		ref	
	T2	3.362(2.380-4.750)	<0.001	2.830(1.950-4.105)	<0.001	1.128(1.006-1.265)	0.039	1.167(1.030-1.323)	0.015
	T3	8.863(6.717-11.693)	<0.001	4.111(2.742-6.163)	<0.001	1.677(1.526-1.843)	<0.001	1.246(1.061-1.463)	0.007
	T4	91.998(71.220-118.838)	<0.001	14.248(8.974-22.622)	<0.001	8.020(7.306-8.804)	<0.001	2.589(2.090-3.208)	<0.001
N stage	N0	ref		ref		ref		ref	
	N1	4.326(3.735-5.012)	<0.001	1.961(1.612-2.385)	<0.001	1.649(1.525-1.782)	<0.001	1.451(1.309-1.609)	<0.001
M-stage	M0	ref		ref		ref		ref	
	M1	50.426(43.883-57.943)	<0.001	5.897(4.785-7.268)	<0.001	15.305(13.911-16.838)	<0.001	3.591(3.094-4.167)	<0.001
Multifocality	No	ref		ref		ref		ref	
	Yes	0.976(0.837-1.139)	0.760	0.871(0.731-1.038)	0.122	0.893(0.827-0.964)	0.014	0.968(0.890-1.053)	0.454
Extension	No	ref		ref		ref		ref	
	Yes	13.542(11.516-15.924)	<0.001	1.607(0.120-2.306)	0.010	2.783(2.583-2.998)	<0.001	1.177(0.985-1.408)	0.073
Radiation	None or refused	ref		ref		ref		ref	
	Radiation Beam or Rdioactive implants	16.161(13.561-19.261)	<0.001	2.223(1.734-2.851)	<0.001	4.318(3.817-4.884)	<0.001	1.222(1.041-1.461)	0.015
	Radioisotopes or Radiation beam+ isotopes/implants	0.901(0.769-1.055)	0.195	0.738(0.603-0.904)	0.003	0.599(0.555-0.646)	<0.001	0.670(0.611-0.735)	<0.001
Surgery	Biopsy	ref		ref		ref		ref	
	Lobectomy	0.036(0.027-0.048)	<0.001	0.491(0.331-0.728)	<0.001	0.091(0.080-0.103)	<0.001	0.315(0.266-0.372)	<0.001
	Subtotal or near-total thyroidectomy	0.081(0.059-0.112)	<0.001	0.699(0.448-1.091)	0.115	0.095(0.079-0.114)	<0.001	0.335(0.269-0.418)	<0.001
	Total thyroidectomy	0.048(0.041-0.056)	<0.001	0.525(0.389-0.707)	<0.001	0.069(0.063-0.076)	<0.001	0.293(0.252-0.340)	<0.001

### Adjusting for patient characteristics using PSM

**Figure 1:**
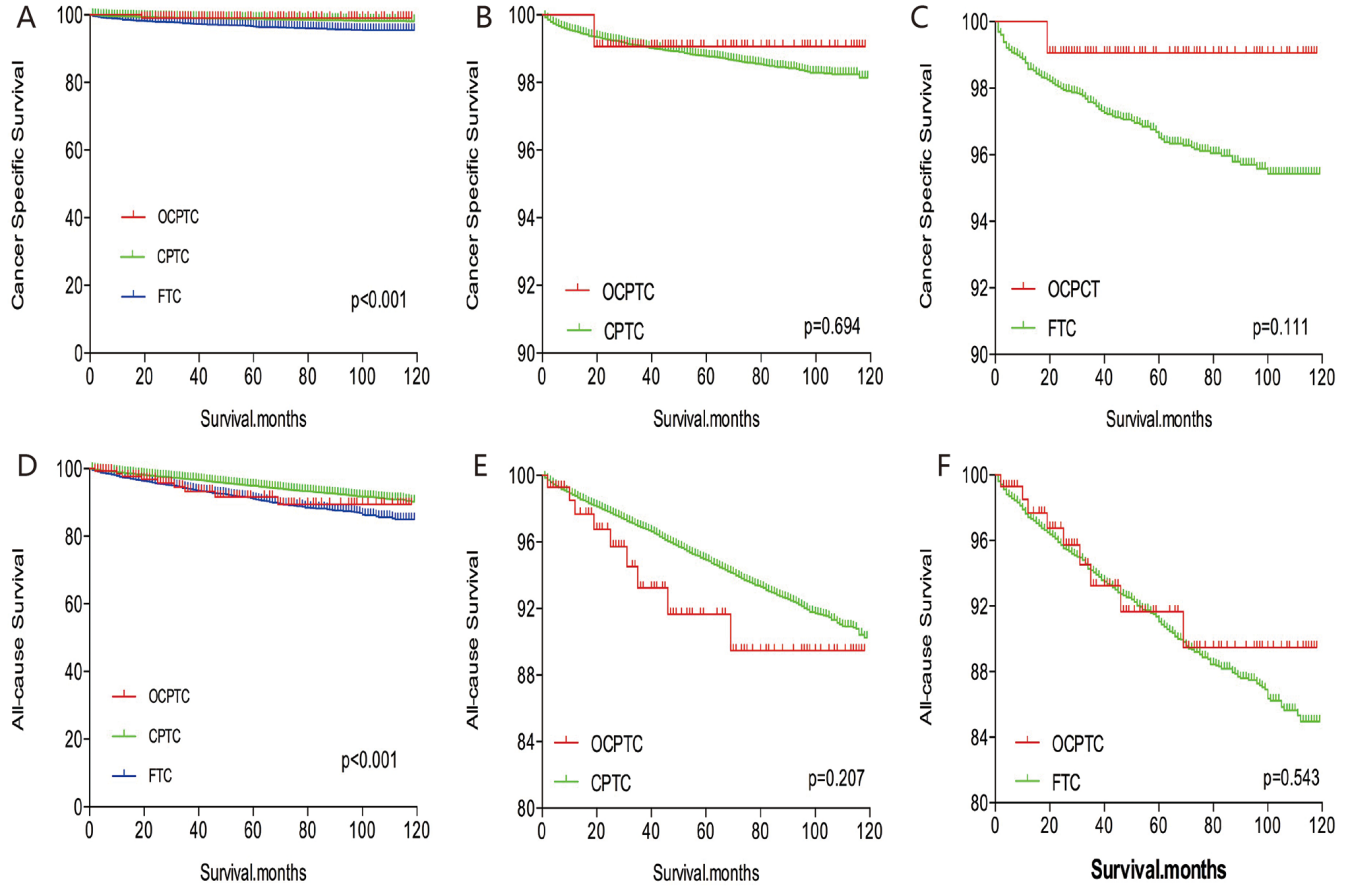
Kaplan Meier curves among patients stratified by subtype for cancer-specific mortality **(A, B, C)** and all-cause mortality **(D, E, F)**.

**Figure 2:**
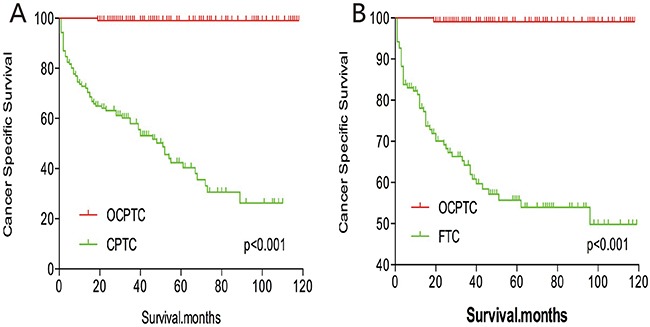
Kaplan Meier curves of cancer-specific mortality for matched subtype pairs Age, sex and race matching between OCPTC and CPTC **(A)**, OCPTC and FTC **(B)** patients.

**Figure 3:**
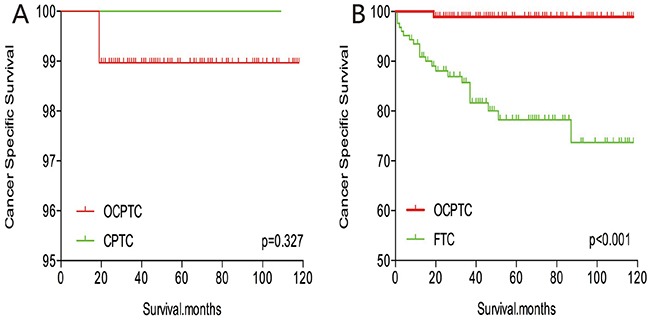
Kaplan Meier curves of cancer-specific mortality for matched Subtype pairs Age, sex, race, T/N/M stage, multifocality, extension matched between OCPTC and CPTC **(A)**, OCPTC and FTC **(B)** patients.

**Figure 4:**
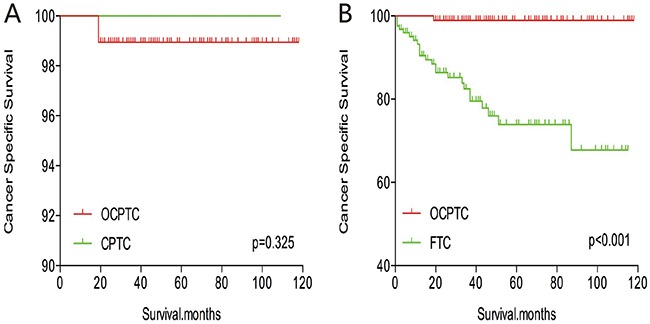
Kaplan Meier curves of cancer-specific mortality for matched Subtype pairs Age, sex, race, T/N/M stage, multifocality, extension and radiation treatment matched between OCPTC and CPTC **(A)**, OCPTC and FTC **(B)** patients.

**Figure 5:**
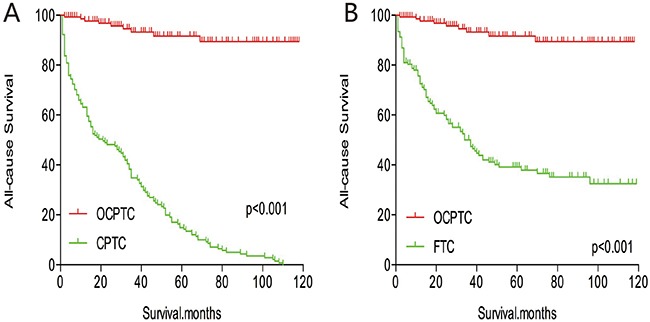
Kaplan Meier curves of all-cause mortality for matched Subtype pairs Age, sex and race matching between OCPTC and CPTC **(A)**, OCPTC and FTC **(B)** patients.

**Figure 6:**
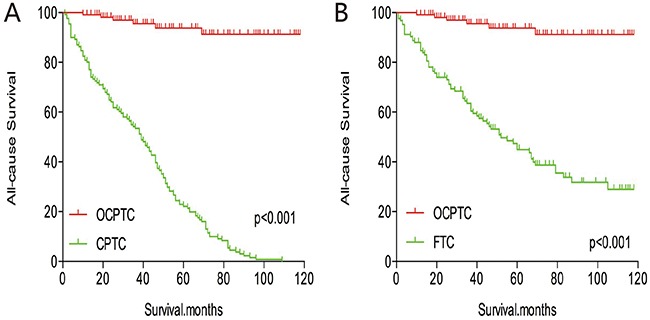
Kaplan Meier curves of all-cause mortality for matched Subtype pairs Age, sex, race, T/N/M stage, multifocality, extension matching between OCPTC and CPTC **(A)**, OCPTC and FTC **(B)** patients.

**Figure 7:**
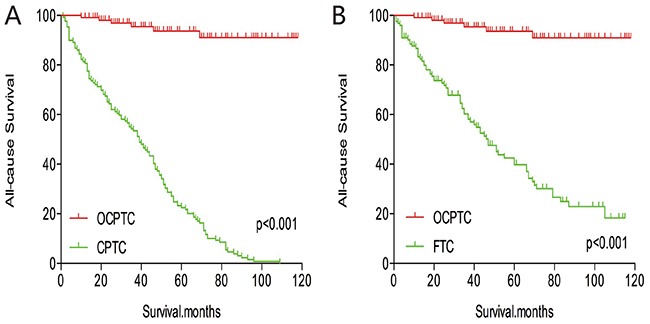
Kaplan Meier curves of all-cause mortality for matched Subtype pairs Age, sex, race, T/N/M stage, multifocality, extension and radiation treatment matching between OCPTC and CPTC **(A)**, OCPTC and FTC **(B)** patients.

The cancer-specific mortality rate of patients with OCPTC was similar to that of patients with CPTC and FTC (p = 0.694 and 0.111, respectively). The all-cause mortality rate of patients with OCPTC was also similar to patients with CPTC and FTC [p = 0.207 and 0.543, respectively; (Figures 1A-1F)]. To minimize selection bias, propensity scored matching analysis was performed for age, sex, race, T/N/M stage, multifocality, extension, and radiation treatment approaches. In the survival analysis, patients with OCPTC had a better prognosis for cancer-specific mortality compared to patients with CPTC and FTC (p <0.001 for both, Figures 2A and 2B) after PSM for age, sex, and race. After PSM for age, sex and race, T/N/M stage, multifocality, and extension, there were no significant differences in cancer-specific mortality between OCPTC and CPTC patients (p = 0.327); however, patients with OCPTC were observed to have a better cancer-specific survival than patients with FTC (p < 0.001; Figures 3A-3B). After matching for all influential factors, including radiation treatment, the prognosis for patients with OCPTC was similar to that of those with CPTC, but better than that of patients with FTC (p = 0.325 and p < 0.001, respectively; Figures 4A-4B). In survival analysis for all-cause mortality, OCPTC had a better prognosis compared to CPTC and FTC after matching for age, sex, and race (all p<0.001, Figure 5A-5B). Similar results were obtained after matching for age, sex and race, T/N/M stage, multifocality, extension (Figure 6A-6B). After matching for all influential factors including radiation treatment, CPTC and FTC patients showed a poorer prognosis for all-cause mortality compared to patients with OCPTC (p < 0.001 for all; Figures 7A-7B).

## DISCUSSION

Oxyphilic cells exhibit a characteristic phenotype, which features a finely granular eosinophilic cytoplasm and an increased number of mitochondria in the thyroid ultrastructurally [16]. Oxyphilic cells could be observed in both follicular and papillary carcinomas, originating from the thyroid gland. Furthermore, oxyphilic cells were also reported in non-neoplastic and neoplastic conditions of many other sites, such as salivary, parathyroid, and kidneys [16].

Oxyphilic cell tumors are a relatively rare histological type of differentiate thyroid carcinoma, and they are diagnosed mainly based on surgical specimens and cytology [17]. Negative results were obtained by previous researchers regarding whether oxyphilic cell thyroid carcinomas (OCTC) have a poorer prognosis than CPTC or FTC [18–20]. Kushchayeva *et al*. demonstrated that OCTC had a poorer cause-specific mortality rate than FTC, and suggested that OCTCs should be classified as distinct tumors [19]. Yasuhiro *et al*. also showed that almost 80% of the tumors were cytologically diagnosed as category IV or greater in the Bethesda System for Reporting Thyroid Cytopathology [17]. However, OCPTCs, a histological subtype of oxyphilic cell tumors, were rarely studied due to their low prevalence. Therefore, we compared the prognosis of OCPTC with that of CPTC and FTC, which are the most frequent histological types of thyroid malignancies.

In our study using the SEER database, histological subtype was not an independent factor for cancer-specific and all-cause mortality rates. Before matching risk variates, OCPTC had a prognosis similar to that of CPTC and FTC. The average age of participants with OCPTC was 52.22 years, which is greater than that of CPTC and FTC patients. According to studies, age is an independent risk factor of thyroid cancer-related deaths [13, 21]. In our study, when demographic profiles of patients were matched (Figure 2A, 2B), patients with OCPTC had significantly higher survival compared to CPTC and FTC patients. This implies that if demographic data for OCPTC, CPTC and FTC patients were identical, OCPTC patients would have a better prognosis. Furthermore, after matching for all confounding factors, including clinicopathological features and radiation treatment, OCPTC had a prognosis similar to CPTC, but better than FTC. Surgeons have not yet established the most logical treatment for OCPTC, and our results may provide guidance for clinicians and complement the new American Thyroid Association guidelines.

Many patients with oxyphilic cells are recommended for total thyroidectomy due to the involvement of other lesions in the contralateral lobe or oncocytic neoplasms in the context of a multinodular goiter [22]. However, it is currently unknown whether patients with OCPTC should undergo prophylactic lymph node dissection. In this current study, only 17.5% of patients with OCPTC underwent lymph node metastasis; therefore, we do not recommend prophylactic lymph node dissection for this subgroup of patients unless positive detection of lymph node metastasis by preoperative ultrasonography.

Considerable attention has been directed towards combining morphological and genetic characteristics of thyroid tumors in recent years, and molecular studies have shed light on the role of various oncogenes in different thyroid tumor subsets [23–25]. Novel molecular-based management strategies, such as *RET-PTC*, *RAS*, *BRAF* (V600E), and *TERT* mutations for thyroid nodules and thyroid cancer are the most exciting developments in thyroid-cancer medicine [26].

Carcinomas with oxyphilic cell features typically harbor the genetic alterations associated with the predominant histotype of the lesion. For example, in the Hürthle cell variant of papillary thyroid carcinomas, there is a high prevalence of *RET-PTC* rearrangements and *BRAF* mutations [27–29]. Therefore, some authors suggested that oxyphilic cell tumors should be treated separately due to histopathological and molecular features [30]. The distinction between malignant and benign oxyphilic cell tumors represents a difficult diagnostic challenge, and these molecular tests may provide a new reference for oxyphilic cell tumor diagnosis and assist with treatment decision-making [22].

Our study had several limitations. Firstly, the utilized dataset lacked information regarding recurrence, which may introduce an overestimation bias when designating cancer-specific and all-cause mortality rates. In addition, we haven't added the time variable as a baseline factor for adjustment. Another limitation of this study is that family history, vascular invasion, and other histologic findings were not evaluated or included in our study. Furthermore, molecular markers, such as *BRAF* point mutation and *TERT* promotor point mutations, were not observed in our study or adjusted for in our analyses.

In summary, based on the results of our investigation, we unexpectedly observed that patients diagnosed with OCPTC had a similar prognosis to CPTC patients, but a better prognosis than FTC patients. Our findings may provide a helpful reference for future treatment decision-making.

## MATERIALS AND METHODS

### Ethics statement

This investigation has been conducted in accordance with the ethical standards, according to the Declaration of Helsinki, and according to national and international guidelines. It has been approved by the authors’ institutional review board.

### Study population

We investigated many patients with thyroid cancer, including CPTC, OCPTC, and FTC, from the SEER program. The SEER project is a United States population-based cancer registry that began in 1973, and is supported by both the Centers for Disease Control and Prevention and National Cancer Institute. It contains cancer data, such as the incidence, prevalence, mortality, population-based variables, and primary tumor characteristics (i.e., histological subtype), from multiple geographic regions.

### Data collection and analysis

We examined SEER data from 2004 to 2013, and selected patients who were diagnosed with CPTC, OCPTC, and FTC, as defined by a combination of ICD-O site code of C73.9 (i.e., thyroid, papillary, and/or follicular histology). The diagnosis codes included in the study were: “papillary carcinoma”, “papillary adenocarcinoma”, “Papillary carcinoma, oxyphilic cell”, “follicular adenocarcinoma”, and “papillary & follicular adenocarcinoma”. The inclusion criteria included patients diagnosed with CPTC, OCPTC, and FTC; cases with diagnosed from 2004 to 2013 because from that time patients had information with unified AJCC TNM staging data.. We excluded the cases with other histological types and without follow-up information. Finally, to compare the survival rate among different histological subtypes, 66305 patients were included for analysis. The age, sex, race, T/N/M stage, multifocality, extension, and radiation treatment (i.e., none or refused, external beam radiation therapy, and radioactive I-131 ablation) were evaluated in patients with different histological subtypes.

### Statistical analyses

Patients were followed-up until December 2013. Patient survival curves for thyroid cancer-specific mortality and all-cause mortality were examined by Kaplan-Meier analyses with the log-rank test. To further adjust for potential baseline confounding factors, a PSM analysis was conducted on demographic data, clinicopathological characteristics of thyroid cancer, and treatment approaches. Cox proportional hazards regression analyses were performed to estimate hazard ratios with 95% CIs and to show the magnitude of the effect of different histological subtypes on cancer-specific mortality and all-cause mortality [31]. All p-values were 2-sided, and p-values <.05 were considered significant. Analyses were performed using SPSS version 23.0, Stata/SE version 12 (Stata Corp.), and GraphPad Prism version 6 (GraphPad Software Inc.).

## Abbreviations

CPTC: classic papillary thyroid cancer
CI: confidence interval
FTC: follicular thyroid cancer
OCPTC: oxyphilic cell papillary thyroid carcinoma
PSM: propensity score matching
PTC: papillary thyroid cancer
SEER: Surveillance, Epidemiology, and End Results

## References

[1] Haugen BR, Alexander EK, Bible KC, Doherty GM, Mandel SJ, Nikiforov YE, Pacini F, Randolph GW, Sawka AM, Schlumberger M, Schuff KG, Sherman SI, Sosa JA (2015). American Thyroid Association Management Guidelines for Adult Patients with Thyroid Nodules and Differentiated Thyroid Cancer: The American Thyroid Association Guidelines Task Force on Thyroid Nodules and Differentiated Thyroid Cancer. Thyroid.

[2] Chen W, Zheng R, Baade PD, Zhang S, Zeng H, Bray F, Jemal A, Yu XQ, He J (2016). Cancer statistics in China, 2015. CA Cancer J Clin.

[3] Simard EP, Ward EM, Siegel R, Jemal A (2012). Cancers with increasing incidence trends in the United States: 1999 through 2008. CA Cancer J Clin.

[4] Colonna M, Uhry Z, Guizard AV, Delafosse P, Schvartz C, Belot A, Grosclaude P (2015). Recent trends in incidence, geographical distribution, and survival of papillary thyroid cancer in France. Cancer Epidemiol.

[5] Mao Y, Xing M (2016). Recent incidences and differential trends of thyroid cancer in the USA. Endocr Relat Cancer.

[6] Liu Z, Zeng W, Chen T, Guo Y, Zhang C, Liu C, Huang T (2017). A comparison of the clinicopathological features and prognoses of the classical and the tall cell variant of papillary thyroid cancer: a meta-analysis. Oncotarget.

[7] Wang X, Cheng W, Liu C, Li J (2016). Tall cell variant of papillary thyroid carcinoma: current evidence on clinicopathologic features and molecular biology. Oncotarget.

[8] Hang JF, Westra WH, Cooper DS, Ali SZ (2017). The impact of noninvasive follicular thyroid neoplasm with papillary-like nuclear features on the performance of the Afirma gene expression classifier. Cancer.

[9] Russo M, Malandrino P, Moleti M, Vermiglio F, Violi MA, Marturano I, Minaldi E, Vigneri R, Pellegriti G, Regalbuto C (2017). Tall cell and diffuse sclerosing variants of papillary thyroid cancer: outcome and predicting value of risk stratification methods. J Endocrinol Invest.

[10] Morandi L, Righi A, Maletta F, Rucci P, Pagni F, Gallo M, Rossi S, Caporali L, Sapino A, Lloyd RV, Asioli S (2017). Somatic mutation profiling of hobnail variant of papillary thyroid carcinoma. Endoc Relat Cancer.

[11] Hod R, Bachar G, Sternov Y, Shvero J (2013). Insular thyroid carcinoma: a retrospective clinicopathologic study. Am J Otolaryngol.

[12] Lim H, Devesa SS, Sosa JA, Check D, Kitahara CM (2017). Trends in Thyroid Cancer Incidence and Mortality in the United States, 1974-2013. JAMA.

[13] Adam MA, Thomas S, Hyslop T, Scheri RP, Roman SA, Sosa JA (2016). Exploring the Relationship Between Patient Age and Cancer-Specific Survival in Papillary Thyroid Cancer: Rethinking Current Staging Systems. J Clin Oncol.

[14] Ganly I, Ricarte Filho J, Eng S, Ghossein R, Morris LG, Liang Y, Socci N, Kannan K, Mo Q, Fagin JA, Chan TA (2013). Genomic dissection of Hurthle cell carcinoma reveals a unique class of thyroid malignancy. J Clin Endocrinol Metab.

[15] Goffredo P, Roman SA, Sosa JA (2013). Hurthle cell carcinoma: a population-level analysis of 3311 patients. Cancer.

[16] Allia E, Cassoni P, Marrocco T, Volante M, Bussolati B, Wong M, Clark OH, Papotti M (2003). Oxyphilic and non-oxyphilic thyroid carcinoma cell lines differ in expressing apoptosis-related genes. J Endocrinol Invest.

[17] Ito Y, Hirokawa M, Miyauchi A, Kihara M, Yabuta T, Masuoka H, Fukushima M, Higashiyama T, Kobayashi K, Miya A (2016). Diagnosis and surgical indications of oxyphilic follicular tumors in Japan: Surgical specimens and cytology. Endocrine J.

[18] Bishop JA, Wu G, Tufano RP, Westra WH (2012). Histological patterns of locoregional recurrence in Hurthle cell carcinoma of the thyroid gland. Thyroid.

[19] Kushchayeva Y, Duh QY, Kebebew E, D’Avanzo A, Clark OH (2008). Comparison of clinical characteristics at diagnosis and during follow-up in 118 patients with Hurthle cell or follicular thyroid cancer. Am J Surg.

[20] Haigh PI, Urbach DR (2005). The treatment and prognosis of Hurthle cell follicular thyroid carcinoma compared with its non-Hurthle cell counterpart. Surgery.

[21] Oh CM, Jung KW, Won YJ, Shin A, Kong HJ, Lee JS (2015). Age-Period-Cohort Analysis of Thyroid Cancer Incidence in Korea. Cancer Res Treat.

[22] Pisanu A, Sias L, Uccheddu A (2004). Factors predicting malignancy of Hurthle cell tumors of the thyroid: influence on surgical treatment. World J Surg.

[23] Xing M (2013). Molecular pathogenesis and mechanisms of thyroid cancer. Nature.

[24] Xing M, Alzahrani AS, Carson KA, Shong YK, Kim TY, Viola D, Elisei R, Bendlova B, Yip L, Mian C, Vianello F, Tuttle RM, Robenshtok E (2015). Association between BRAF V600E mutation and recurrence of papillary thyroid cancer. J Clin Oncol.

[25] Shen X, Liu R, Xing M (2017). A six-genotype genetic prognostic model for papillary thyroid cancer. Endocr Relat Cancer.

[26] Xing M, Haugen BR, Schlumberger M (2013). Progress in molecular-based management of differentiated thyroid cancer. Lancet.

[27] Maximo V, Preto A, Crespo A, Rocha AS, Machado JC, Soares P, Sobrinho-Simoes M (2004). Core I gene is overexpressed in Hurthle and non-Hurthle cell microfollicular adenomas and follicular carcinomas of the thyroid. BMC Cancer.

[28] Soares P, Trovisco V, Rocha AS, Lima J, Castro P, Preto A, Maximo V, Botelho T, Seruca R, Sobrinho-Simoes M (2003). BRAF mutations and RET/PTC rearrangements are alternative events in the etiopathogenesis of PTC. Oncogene.

[29] Cheung CC, Ezzat S, Ramyar L, Freeman JL, Asa SL (2000). Molecular basis off hurthle cell papillary thyroid carcinoma. J Clin Endocr Met.

[30] Volante M, Papotti M, Gugliotta P, Migheli A, Bussolati G (2001). Extensive DNA fragmentation in oxyphilic cell lesions of the thyroid. J Histochem Cytochem.

[31] Spruance SL, Reid JE, Grace M, Samore M (2004). Hazard ratio in clinical trials. Antimicrob Agents Chemother.

